# Outcomes of Women Undergoing Mastectomy for Unilateral Breast Cancer Who Elect to Undergo Contralateral Mastectomy for Symmetry: A Systematic Review

**DOI:** 10.1245/s10434-023-14294-6

**Published:** 2023-09-25

**Authors:** Cora Griffin, Katherine Fairhurst, Imogen Stables, Sam Brunsden, Shelley Potter

**Affiliations:** 1 Bristol Medical School, University of Bristol, Bristol, UK; 2 King’s College Hospital NHS Foundation Trust, London, UK; 3 Flat Friends, London, UK; 4 Bristol Breast Care Centre, North Bristol NHS Trust, Bristol, UK

**Keywords:** Breast cancer, Mastectomy, Symmetry, Patient-reported outcomes, Complications

## Abstract

**Background:**

Breast reconstruction (BR) is routinely offered to restore symmetry after mastectomy for breast cancer. Not all women, however, may want reconstructive surgery. A contralateral mastectomy (CM) to achieve “flat symmetry” can be an excellent alternative, but surgeons are often reluctant to offer this procedure. This systematic review aimed to summarize the available evidence regarding the outcomes of CM as the first step to developing guidelines in this area.

**Methods:**

PubMed, MEDLINE, CINAHL and PsycINFO were searched to identify primary research studies, published in English between 1 January 2000 and 30 August 2022, evaluating clinical or patient-reported outcomes for women who underwent a CM without reconstruction after a mastectomy for unilateral breast cancer. Simple descriptive statistics summarized quantitative data, and content analysis was used for qualitative data.

**Results:**

The study included 15 studies (13 quantitative, 1 qualitative, and 1 mixed-methods) evaluating outcomes for at least 1954 women who underwent a bilateral mastectomy without reconstruction (BM) after unilateral breast cancer. The risk of surgical complications after BM was higher than after unilateral mastectomy without reconstruction (UM) but significantly less than after BR. Satisfaction with the decision for BM was high in all the studies. Key themes relating to flat denial, stigma, and gender-based assumptions were identified.

**Conclusion:**

Women electing to undergo BM reported high levels of satisfaction with their decision and complication rates similar to those for UM. Further study is needed to comprehensively explore the outcomes for women seeking BM, but these data should give surgeons confidence to offer the procedure as an alternative option for symmetry after unilateral mastectomy for breast cancer.

*Registration*: This systematic review was prospectively registered on the PROSPERO database (CRD42022353689).

Breast cancer is diagnosed for more than 55,000 women every year in the UK, 40% of whom undergo mastectomy.^1,2^ Breast reconstruction is routinely offered to restore symmetry,^3^ but complication rates are high,^4,5^ and the long-term outcomes can be poor.^6,7^ Furthermore, not all women want (or are suitable for) reconstructive surgery.

Many women, however, wish to be symmetrical without breast reconstruction. For some, an external prosthesis is acceptable, but others report feeling unbalanced^8,9^ and wish to be symmetrical when unclothed. For this group, a contralateral mastectomy (CM) providing “flat symmetry” is becoming an increasingly popular option.

Whereas clear guidelines exist for offering breast reconstruction routinely to all women,^3^ no recommendations currently exist regarding the provision of contralateral symmetrizing mastectomy (CSM). In a survey of 207 women by the Flat Friends group, less than one third were given information about going fully flat, with only 11% offered CSM as an option. More than half of these women felt ill-informed of the surgical options available after mastectomy,^10^ reflecting inequalities in the provision of care.^9^

One reason for the difficulties that women have accessing CSM may be the confusing and often misleading terminology associated with the procedure, which is often referred to as a contralateral “prophylactic” or “risk-reducing’ mastectomy,”^11^ even when women are seeking surgery to achieve flat symmetry. Contralateral mastectomy to reduce risk for those with unilateral breast cancer but no high genetic or familial risk for breast cancer is strongly discouraged by the UK professional associations^12^ due to lack of oncologic benefit. In addition, clinicians express concerns that women may regret their decision to have CM and may later seek bilateral reconstruction, a challenging and expensive procedure.^13^ Surgeons are therefore reluctant to offer this option, and many women describe a battle to be “allowed” a CSM, often having to follow complex local pathways, including the need for psychological assessment before surgery.^14^

Additionally, different areas of the UK have variable policies regarding the funding for CSM, with some refusing to fund it despite routine funding for other symmetrizing procedures, including contralateral breast reduction after mastectomy. Funding constraints are often cited as the main rationale for not offering CSM despite its lower cost compared with breast reconstruction,^9,15^ although clinicians’ reluctance to offer women this option is likely to be complex and multifactorial.^14^

More research is needed to improve the experiences and outcomes of women seeking CSM. This systematic review aimed to identify and summarize the evidence regarding clinical and patient-reported outcomes for women electing to undergo contralateral simple mastectomy after unilateral mastectomy for breast cancer as the first step toward developing evidence-based guidelines and a standardized patient pathway.

## Methods

This systematic review was prospectively registered in the PROSPERO database (CRD42022353689).

### Search Strategy and Data Sources

A comprehensive literature search was undertaken in MEDLINE, PubMed, CINAHL, and PsycINFO using a search strategy developed in collaboration with a specialist subject librarian. The search terms included “flat closure” OR “contralateral mastectomy” OR “contralateral risk reducing mastectomy” OR “prophylactic mastectomy/” OR “contralateral prophylactic mastectomy” OR “double mastectomy” OR “bilateral mastectomy” OR “preventive mastectomy” OR “risk-reducing mastectomy.” The search was limited to human studies, published in English between 1 January 2000 and 30 August 2022 to ensure that results reflected current practice.

### Study Inclusion and Exclusion Criteria

All quantitative and qualitative studies published in full in English, evaluating the clinical or patient-reported outcomes or experiences of women undergoing CM without reconstruction after a unilateral mastectomy for primary or locally recurrent breast cancer were eligible for inclusion in the study. Because CM for symmetry is often mislabeled as “contralateral prophylactic mastectomy” (CPM) or “contralateral risk-reducing mastectomy” (CRRM), the review included data on all women undergoing bilateral mastectomy for a unilateral breast cancer without reconstruction. The CM could be performed either at the time of the index mastectomy or as a separate procedure.

The study excluded abstracts, letters, and conference reports due to difficulties evaluating incomplete information. Reviews and opinion pieces also were excluded. Snowball searching of reference lists of relevant papers and reviews were used to identify any additional potentially relevant publications.

Abstracts were imported to Rayyan review management software^16^ and de-duplicated. Two independent reviewers (C.G., I.S.) screened each abstract according to prespecified inclusion criteria (Table 1). Studies that did not specifically state their inclusion and exclusion criteria were included for full-text review. Papers exclusively reporting outcomes for high-risk women (e.g., with a family history of breast cancer) were excluded because they would not include the CSM cohort. Discrepancies were resolved by discussion with the wider study team (S.P., K.F.), with full texts obtained and reviewed if necessary.

**Table 1:** Inclusion and exclusion criteria

Inclusion criteria	Exclusion criteria
Unilateral breast cancer Women older than 18 years Group that had bilateral mastectomy without reconstruction	All women in the study underwent reconstruction All women have a family history of breast cancer or BRCA mutation No separate analysis of the cohort that had bilateral mastectomy without reconstruction No outcomes reported after surgery Not primary research study

### Data Extraction

Data were extracted by one reviewer (C.G.) using a data collection form iteratively developed in Microsoft Excel (Redmond, WA) for the review, with approximately 10% double-extracted by another member of the team (K.F.) to ensure rigor. The data extracted included study details (author, year of publication, number of participants, study design), type of outcomes reported, key findings, and limitations. Outcomes and outcome-related data were extracted verbatim and summarized in tables.

### Statistical Analysis

Simple summary statistics were used for quantitative data, and content analysis^17^ was used for qualitative data.

## Results

### Study Selection

**Fig. 1:**
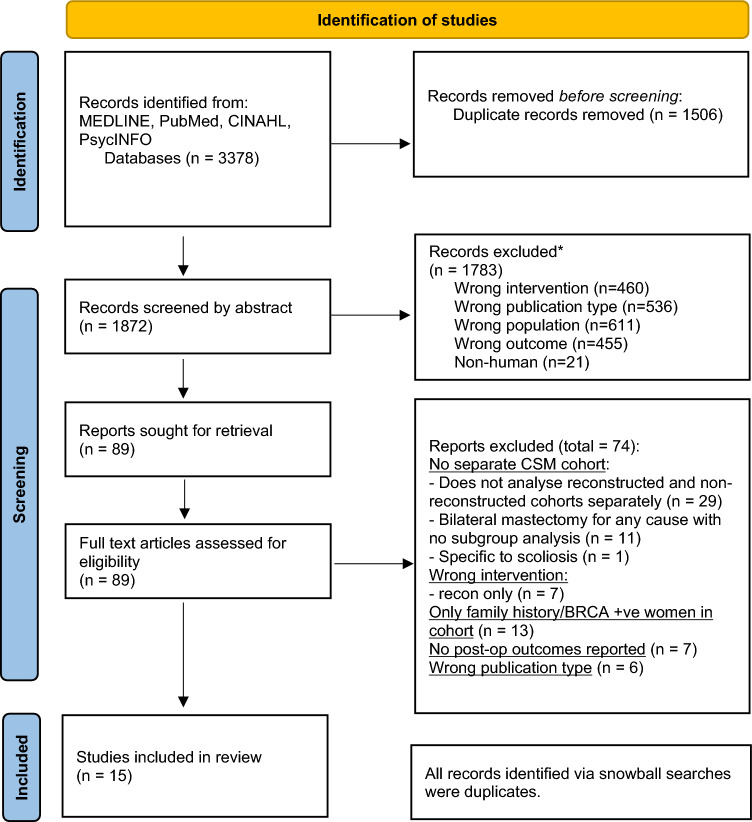
PRISMA diagram

The search, performed 30 August 22, identified 3378 abstracts, 1872 of which remained after de-duplication. After abstract screening, 89 full-text articles were reviewed. Because 40 of these studies did not specifically report the outcomes for the cohort of interest, they were excluded due to inability to extract relevant outcome data. Other studies were excluded because they reported outcomes only for women who had bilateral reconstruction (*n* = 7), included only high-risk women (*n* = 13), did not report outcomes of interest (*n* = 7), or did not report the results of primary research (*n* = 6) (Fig. 1). The review included 15 papers reporting outcomes from 13 quantitative studies,^18–30^ 1 qualitative study,^31^ and 1 mixed-methods study^32^ (Fig. 1; Table 2).

**Table 2:** Summary of papers included in the review (*n* = 15)

Author (year of publication)	Outcome	Country	Study design	Total patients in study (*n*)	CSM/BM (*n*)	BM + R (*n*)	UM (*n*)	UM + R (*n*)	Limitations
Baker (2021)^32^	Patient-reported	US, CA, GB, AUS	Cross-sectional, qualitative and quantitative	931	748	–	183	–	Included BM for bilateral cancer and prophylaxis.
Brown (2018)^31^	Patient-reported	US	Qualitative	16	16	–	–	–	Does not mention BRCA or family history exclusion NB: part of larger study of 68 participants, only BM cohort (*n* = 16) analyzed in this paper Extrapolation of qualitative data from this paper is limited as only SGM people were included
Delierel (2021)^18^	Patient-reported	US	Cross-sectional, quantitative	1525	106	414	109	219	Included cohort with family history of BC 677 underwent BCS
Eck (2014)^19^	Clinical	US	Retrospective cohort, quantitative	352	39	108	114	91	
Huang (2018)^21^	Clinical	US	Retrospective cohort, quantitative	471	17	259	82	113	Included women with BRCA mutations
Huang (2020)^20^	Patient-reported	US	Cross-sectional, quantitative	55	NS	NS	NS	NS	Does not mention BRCA or family history exclusion
Huang (2018)^22^	Patient-reported	US	Cross-sectional, quantitative	94	NS (BM ± recon = 52)	NS	NS (UM ± recon = 42)	NS	Few BM patients without reconstruction (21.3% of total participants had no reconstruction)
Hwang (2016)^23^	Patient-reported	US	Cross-sectional, quantitative	3977	326	1270	889	1490	Does not mention BRCA or family history exclusion
Lim (2021)^24^	Patient-reported	CA	Prospective cohort, quantitative	475	16	75	113	69	BREAST-Q score differences may not be clinically significant 202 underwent unilateral lumpectomy
Miller (2013)^25^	Clinical	US	Retrospective cohort, quantitative	600	21	188	177	214	Does not mention BRCA or family history exclusion
Osman (2013)^26^	Clinical	US	Retrospective cohort, quantitative	4219	497	–	3722	–	Data were extracted by coding from large database
Pinell-White (2014)^27^	Clinical	US	Retrospective cohort, quantitative	446	28	146	99	173	Included cohort with family history of BC. Only 2 complications in BM group
Rosenberg (2020)^28^	Patient-reported	US	Prospective cohort, quantitative	826	NS (BM ± recon = 375)	NS	NS (UM ± recon = 197)	NS	Included cohort with family history of BC and BRCA mutation
Schroeder (2020)^29^	Clinical	US	Retrospective cohort, quantitative	12,959	95	1289	6223	5352	Does not mention BRCA or family history exclusion
Sharpe (2014)^30^	Clinical	US	Retrospective cohort, quantitative	39,0712	NS (BM ± recon = 75437)	NS	NS (UM ± recon = 315278)	NS	Does not mention BRCA or family history exclusion. Data extracted by coding from large database

*CSM* contralateral symmetrizing mastectomy, *BM* bilateral mastectomy, *R* reconstruction, *UM* unilateral mastectomy, *US* United States, *CA* Canada, *GB* Great Britain, *AUS* Australia, *NB* nota bene (please note) *BC* breast cancer, *NS* not stated, *SGM* sexual and gender minority, *BCS* breast-conserving surgery

From these studies, the following four cohorts of women were identified: women undergoing bilateral mastectomy without reconstruction (BM), women who underwent bilateral mastectomy with reconstruction (BM + R), women who had unilateral mastectomy without reconstruction (UM), and women who underwent unilateral mastectomy with reconstruction (UM + R). Overall, at least 1954 patients underwent BM, but not all the studies documented the number of women in this subgroup.

### Clinical Outcomes

Seven studies reported the clinical outcomes of UM and BM (Table 3). Six of these seven studies reported clinical outcomes for UM and BM both with and without reconstruction,^19,21,25,27,29,30^ and the remaining study reported outcomes only for UM and BM without reconstruction.^26^ Only two studies reported the timing of the CM,^19,24^ and all the procedures were performed at the time of the index mastectomy.

**Table 3:** Summary of clinical outcomes from included studies (*n* = 7)

Author	Total patients in study	% Of cohort with any complication (if given in study)				Conclusions
		BM/CSM	UM^a^	UM + R	BM + R	
Eck (2014)^19^	352	38% (*n* = 15)	20% (*n* = 23) Reference	27% (*n* = 25)	29% (*n* = 31)	**Reconstruction doubled risk** for any complication and **quadrupled risk** for major complications. BM did not confer significantly more complications or a higher reoperation rate. Nearly half of CSM complications on contralateral side
Huang (2018)^21^	471	–	–	–	–	Multivariate analysis: BM not associated with higher complications or reoperation independent of reconstruction. **Longer hospital stay for BM patients**
Miller (2013)^25^	600	42.9% **OR 1.5**	21.5% Reference	–	–	Multivariate analysis: **BM conferred an OR of 1.5** for any complication and **autologous reconstruction an OR of 2.6**. Major complications conferred an OR of **2.7** for BM and **5.9** for autologous reconstruction. Half of CSM complications on the contralateral side
Osman (2013)^26^	4219	**OR 1.9**	Reference	–	–	**Wound and infectious complications higher,** not respiratory, thromboembolic, renal, neurologic, cardiac, and bleeding complications
Pinell-White (2014^27^	446	7% (*n* = 2) **OR 2.11**	1% (*n* = 1) Reference	35.0% (*n* = 73)	50.3% (*n* = 56)	Multivariate analysis: **reconstruction conferred a 20-fold increase** in complications
Schroeder (2020)^29^	12959	12.6% OR 1.18	12.1% Reference	–	30.7% **OR 3.91**	Overall no difference in complication or reoperation rates with or without reconstruction. For **incidence of further breast-related procedures, CSM** confers an **OR of 0.76 versus UM and BM + R an OR of 22.81**
Sharpe (2014)^30^	390712	–	–	–	–	2-Day **inpatient stay** for CSM versus 1 day for UM. No difference in readmission rates and 30-day mortality with CSM versus UM

*BM* bilateral mastectomy, *CSM* contralateral symmetrizing mastectomy, *UM* unilateral mastectomy, *R* reconstruction, *OR* odds ratio (given for after multivariate analysis where available)

*n* number of patients in cohort with any complication, *CSM* contralateral symmetrising mastectomy

Statistically significant findings are in bold

^a^UM is used as a reference value in all original studies with odds ratios comparing the other cohort’s odds of any complication to UM

The impact of bilateral surgery on the complication rates was inconsistent. Three studies^25–27^ reported higher rates of complications in the BM group than in the UM group, with odds ratios (ORs) ranging from 1.5 (95% confidence interval [CI] 1.0–2.3) to 2.1 (95% CI, 1.3–3.5) for any complication and 2.7 (95% CI 1.4–5.2), whereas the remaining four studies suggested that bilateral surgery did not have a significant impact on complication rates.^19,21,29,30^ Two studies reported the laterality of complications and found that approximately half were on the non-cancer (contralateral) side.^19,25^ Two studies noted longer inpatient stays (average, 2 days vs. 1 day) for women who had bilateral surgery.^21,30^ Three studies investigated reoperation rates and found no significant difference between bilateral and unilateral mastectomies.^19,21,29^ The most common complications for BM were hematoma, seroma, and general surgical complications (pneumonia, urinary tract infection [UTI]).^19,26^

Breast reconstruction was associated with significantly higher rates of complications in all the studies.^19,25,27,29^ One study reported that reconstruction was independently associated with any complication (OR, 2.6) as well as major complications (OR, 5.9).^25^ Pinell-White et al.^27^ reported that the risk of any complication for BM + R was 20 times higher than for CSM. In the study by Schroeder et al.,^29^ BM+R had an OR of 3.91 for any complication versus UM, whereas BM alone showed no difference (30% rate for UM vs. 12% rate for BM). Furthermore, BM + R conferred an OR of 22.81 for incidence of further breast-related procedures (vs. UM), whereas CSM resulted in a decrease in the odds of having further surgery (OR, 0.76).

### Patient-Reported Outcomes

Seven studies described the patient-reported outcomes of UM and BM (Table 4).

**Table 4:** Summary of patient-reported outcomes from included studies (*n* = 7)

PROM	Author	Effect of contralateral symmetrizing mastectomy on patient-reported outcomes	Conclusion
	Measurement tool		
Satisfaction with decision (SWD)	Baker 2020^32^	% satisfied with results Odds ratios for factors associated with satisfaction < 3/5 on Likert scale (lower OR = higher satisfaction): Low surgeon support for decision/flat denial (OR 3.85, *p* < 0.001) BMI > 30 (OR 2.74, *p* < 0.001) UM (OR 1.99, *p* < 0.002). First choice of surgery is CSM (OR 0.63, *p* = 0.049) Surgeon with exclusive breast practice (OR 0.56, *p* < 0.002) Adequate information given for surgical options (OR 0.48, *p* < 0.0001)	Women are satisfied with CSM Good overall satisfaction for all breast surgery Surgeon factors and complications with reconstruction influence satisfaction Trend toward higher satisfaction with reconstruction in Huang’s study but no difference in Deliere’s Patients should be encouraged to speak to others about their experiences to help with decision-making
	Likert scale questionnaire (3-item)		
	Deliere 2021^18^	% of participants with high decision regret (top quartile on decision regret scale): BM: 10% BM + R, no surgical complications: 9% BM + R with surgical complication: 28% (*p* < 0.0001 for difference between BM procedures on chi-square test) UM: 27.5% UM + R, no surgical complications: 23% UM + R with surgical complication: 38.6% (*p* = 0.0006 for difference between UM procedures on chi-square test)	
	Decision Regret Scale^a^ (5-item)		
	Huang 2020^20^	Mean SWD for BM ± reconstruction (whole cohort) was 4.85/5 Factors associated with higher SWD: Use of other cancer patients’ experiences to help decision-making (95.2% SWD vs. 61.8% without using others’ experiences, OR 9.4, *p* = 0.049). Autologous reconstruction versus no reconstruction (OR 5.83) Implant reconstruction versus no reconstruction (OR 4.01) (*p* = 0.306 for difference between BM, BM + autologous reconstruction and BM + implant reconstruction)	
	Satisfaction with Decision scale^a^ (6-item)		
Body image	Huang 2018^22^	No difference between groups, reconstruction not associated with better body image.	Mixed results, vary by questionnaire used Questionnaires may not be best placed to explore complex topic of body image after CSM
	BIBCQ^a^		
	Rosenberg 2020^28^	BM without reconstruction had highest% of participants with “a fair amount” of issues (responses of at least “a fair amount” on CARES scale) for each subscale item. Groups compared: BM, BM + R, UM, UM + R, breast-conserving surgery. (*p* < 0.002 for differences between groups on chi-square test)	
	CARES^a^		
	Hwang 2016^23^	CSM versus UM (without reconstruction): no difference BREAST-Q scores (out of 100, higher score means better outcome): BM: 54, UM: 54.7, BM + R:62, UM + R: 59.9 On multivariate analysis, BM (± reconstruction) and reconstruction were independently associated with better scores.	
	“Satisfaction With Breasts” scale of BREAST-Q^a^		
	Lim 2021^24^	Multivariate analysis: immediate reconstruction conferred score increase of 7.15 (*p* = 0.002) UM±R (vs BM±R) conferred score increase of 8.04 (*p* = 0.008)	
	“Satisfaction With Breasts” scale of BREAST-Q^a^		
QoL	Huang 2018^22^	No difference between groups, reconstruction not associated with better QoL.	QoL not different between surgical groups
	FACT-B^a^		
	Hwang 2016^23^	CSM versus UM (without reconstruction): no difference. BREAST-Q scores (out of 100, higher score means better outcome): BM: 69.1, UM: 69.3, BM + R: 71.7, UM + R: 73.9 On multivariate analysis, BM (± reconstruction) and reconstruction were independently associated with better scores.	
	“Psychosocial Well-Being” scale of BREAST-Q^a^		
	Lim 2021^24^	Multivariate analysis: immediate reconstruction conferred score increase of 6.60 (*p* = 0.007) UM±R (vs. BM±R) conferred score increase of 3.24 (*p* = 0.21)	
	“Psychosocial Well-Being” scale of BREAST-Q^a^		
Sexuality	Hwang 2016^23^	CSM versus UM (without reconstruction): no difference. BREAST-Q scores (out of 100, higher score means better outcome): BM: 39.9, UM: 42.7, BM + R: 48.6, UM + R: 50 On multivariate analysis, reconstruction was independently associated with better scores.	Mixed results Some evidence for negative impact compared with other surgical groups
	“Sexual Well-Being” scale of BREAST-Q^a^		
	Lim 2021^24^	Multivariate analysis: immediate reconstruction conferred score increase of 18.98 (*p* = 0.0001) UM±R (vs. BM±R) conferred score increase of 0.57 (*p* = 0.80)	
	“Sexual Well-Being” scale of BREAST-Q^a^		
	Rosenberg 2020^28^	BM without reconstruction had highest % of participants with “a fair amount” of issues (responses of at least “a fair amount” on CARES-SF scale) for each subscale item. Groups compared: BM, BM + R, UM, UM + R, breast-conserving surgery. (*p* < 0.001 – 0.36 for differences between groups on chi-square test)	
	CARES-SF^a^		
Physical	Hwang 2016^23^	CSM versus UM (without reconstruction): no significant difference in BREAST-Q scores. BREAST-Q scores (out of 100, higher score means better outcome): BM: 75, UM: 76.2, BM + R: 74.5, UM + R: 76.8	No evidence for difference between surgical groups
	“Physical Well-Being” scale of BREAST-Q^a^		
	Lim 2021^24^	Multivariate analysis: immediate reconstruction conferred score increase of 4.07 (*p* = 0.027) UM±R (vs. BM±R) conferred score decrease of 1.33 (*p* = 0.44)	
	“Physical Well-Being” scale of BREAST-Q^a^		
Flat denial (quantitative part of study)	Baker 2020^32^	20.7% felt surgeon did not support their decision to go flat. 22.2% experienced a high level of flat denial (scoring <3/5 points on Likert scale) Two significant factors were associated with flat denial (<3/5 on Likert scale) (lower OR = lower flat denial): Female surgeon (OR 0.59, *p* = 0.001) Surgeon with exclusive breast practice (OR 0.48, *p* < 0.0001)	Many women experienced clinicians who do not support choice of CSM. Female clinicians and those with exclusive breast practice less likely to exhibit flat denial
	3-item (Likert scale) questionnaire		

*PROM* patient-reported outcome measure, *SWD* satisfaction with decision, *CSM* contralateral symmetrising mastectomy, *OR* odds ratio, *BMI* body mass index, *UM* unilateral mastectomy, *BM* bilateral mastectomy, *BM* *+* *R* bilateral mastectomy with reconstruction, *UM* *+* *R* unilateral mastectomy with reconstruction, *BIBCQ* Body Image After Breast Cancer Questionnaire, *CARES* Cancer Rehabilitation Evaluation System, *QoL* quality of life, *FACT-B* Functional Assessment of Cancer Therapy–Breast, *CARES-SF* Cancer Rehabilitation Evaluation System–Short Form

Validated tool

^a^Statistics are given after multivariate analysis where available

#### Satisfaction with Decision

Three studies evaluated patients’ satisfaction with their decision (SWD) to have surgery.^18,20,32^ Although different measures were used, all three studies found that the women were highly satisfied with their decision to have surgery (Table 4). Baker et al.^32^ showed that 74.1% of women were satisfied with BM results and that low satisfaction was associated most strongly with poor surgeon support around decision-making.^32^

Other factors that had a negative impact on patients’ satisfaction with their decision included having a high body mass index (BMI > 30 kg/m^2^) and undergoing UM rather than BM.^32^ High satisfaction was associated with provision of adequate information on surgical options, care provided by a specialist breast surgeon, and BM (“going flat”) as the patient’s first choice of surgery.^32^

In the Deliere et al.^18^ study, BM and BM+R had the lowest proportion of women with high decisional regret (10% for BM and 9% for BM+R), and BM was associated with significantly less regret than UM in the multivariable analysis (OR, 0.40; *P* < 0.001). Complications after reconstructive surgery increased the degree of decisional regret experienced, but was lowest overall among the BM patients of all the surgical types after adjustment for clinical factors.^18^ Huang and Chagpar^20^ demonstrated greater satisfaction with decision among women undergoing BM+R than among those undergoing BM, but this difference was not statistically significant (OR, 5.83 for autologous and 4.01 for implant reconstruction vs. no reconstruction; *p* = 0.306).

#### Body Image

Four studies evaluated body image^22–24,28^ using three validated questionnaires: BREAST-Q: Satisfaction with Breasts’ scale;^23,24^ CARES (Cancer Rehabilitation Evaluation System: 3-item body image subscale),^28^ and BIBCQ (Body Image After Breast Cancer Questionnaire^22^). The results were inconsistent and conflicting (Table 4).

Body image was not affected by the receipt of BM or reconstruction in a study using the BIBCQ scale,^22^ but in a study using the CARES questionnaire, women who had BM reported worse body image outcomes, with the highest percentage of patients having at least “a fair amount” of issues (> 2 on a scale of 0 to 4, with higher values indicating a more severe problem). For example, those with at least “a fair amount” of issues on the subscale item “discomfort with body changes” at the 1-year follow-up evaluation reported rates of 43.8% for BM, 40.6% for BM + R, 34.6% for UM + R, 28.3% for UM, and 25.2% for breast-conserving surgery (*p* < 0.002).^28^ The “Satisfaction With Breasts” (or chest) score on the BREAST-Q questionnaire were lower for women who had UM or BM without reconstruction (BM, 54; UM, 54.7; BM+R, 62; UM + R, 59.9 scores out of 100), but in the multivariate analysis, BM (irrespective of reconstruction) and receipt of reconstruction were independently associated with better scores.^23^ Lim et al.^21^ did not evaluate a separate BM cohort, but performed multivariate analyses with “immediate reconstruction” and “bilateral mastectomy” (irrespective of reconstruction) as variables. Reconstruction improved the “Satisfaction With Breasts” score by 7.15 (*p* = 0.002), whereas BM reduced the score by 8.04 (*p* = 0.008).

#### Quality of Life

Three studies evaluated quality of life^22,24,31^ using validated condition-specific questionnaires: the FACT-B (Functional Assessment of Cancer Therapy–Breast) and the “Psychosocial Well-Being” scale of the BREAST-Q. The FACT-B scores did not differ between the women undergoing BM with reconstruction and those who had BM without reconstruction.^22^ The BREAST-Q “Psychosocial Well-Being” scores were slightly higher for the women who had reconstruction after mastectomy (BM, 69.1; UM, 69.3; BM + R, 71.7; UM + R, 73.9). In the multivariate analysis, BM (irrespective of reconstruction) and receipt of reconstruction were independently associated with better “Psychosocial Well-Being” scores^23^ (Table 4).

#### Sexuality

Three studies evaluated sexual functioning after surgery, again using different instruments^23,24,28^ but with similar findings. In one study using the CARES-SF (Cancer Rehabilitation Evaluation System–Short Form: 3-item sexual subscale) questionnaire, a higher percentage of women who underwent BM without reconstruction reported having “a fair amount” of issues than those who had BM + R, UM, UM + R, or breast conserving surgery (*p* < 0.001–0.36 for differences between groups on the chi-square test).^28^ In a second study, using the BREAST-Q “Sexual Well-Being” scale, women undergoing BM reported the lowest scores (BM, 39.9; UM, 42.7; BM + R, 48.6; UM + R, 50).^23^ In the multivariate analysis, immediate reconstruction improved the sexual well-being score by 18.98 points (*p* = 0.0001).^24^

#### Physical Well-Being

Two studies used the BREAST-Q “Physical Well-Being” scale to report the physical effects of BM. One study showed no differences in scores between the groups (BM, 75; UM, 76.2; BM + R, 74.5; UM + R, 76.8),^28^ but in a multivariable analysis from the second study, immediate reconstruction improved the “Physical Well-Being” score by 4.07 (*p* = 0.027).^24^

#### Flat Denial

Flat denial is defined as an attitude of stigmatizing and not accepting going flat as a valid option, sometimes manifesting in surgical practice with “incomplete resection of soft tissue from the chest . . . result[ing] in suboptimal aesthetic outcomes.”^33^ Baker et al.^32^ used a study-specific 3-item questionnaire to quantify levels of flat denial in their cohort. In their study, 22% of the participants experienced a high level of flat denial. Those with a female surgeon or a surgeon with exclusive breast practice were significantly less likely to experience this (OR, 0.59 and 0.48, respectively).^32^

### Qualitative Themes

The two qualitative papers provided further data to contextualize the quantitative findings (Table 5). These studies also considered women’s motivations for surgery.

**Table 5:** Summary of key themes from qualitative studies (*n* = 2)

Theme	Author	Findings	Conclusions
	Measurement tool		
Motivations for having CSM	Baker 2020^32^	Top motivations: avoiding a foreign body and lower complication rates compared with reconstruction	Women have many different reasons for wanting CSM; they may relate to individual’s identity but cannot make assumptions on motivations
	Brown 2018^31^	Themes related to choosing BM: relationship between participants’ surgical treatment choice and their sexual/gender identity, HCP reactions to this treatment choice, gender-policing and “heterosexism” encountered during and after treatment, and the impact of treatment on participants’ lives and relationships after surviving BC	
Flat denial	Baker 2020^32^	Examples given: ‘‘I was never given the choice of going flat; it was like I was ‘expected’ to have reconstruction . . .” ‘‘I stated multiple times that I intended to stay flat . . . after surgery they told me they left extra skin in case I changed my mind.’’	Flat denial reduces satisfaction Many women experienced biased clinicians who do not support choice of CSM Need for education of HCPs on heteronormative assumptions.
	Brown 2018^31^	Sexual/gender identity helped HCPs understand the choice for CSM. Assumptions or gender bias toward having reconstruction. Strong negative reactions and significant concerns to CSM requests.	
Other themes	Brown 2018^31^	(1) Gender and going flat: treatment choice and feelings about having CSM affected by sexual/gender identity. Lack of support groups/resources for people who aren’t heterosexual (2) HCP reaction to CSM (3) Gender-policing and heterosexism: patient education materials targeted at heterosexual women and focus on appearance. Lack of appropriate clothing for women with CSM (4) Impact of BC on intimacy: some reported positive impact (“I love my scars”), but more reported negative impact (loss of sensation and physical disability). Negative impact as much from medical breast cancer treatments as surgical treatments (5) Life after going flat: questionnaires may assume a negative emotional impact of breast surgery, but participants commented this misses nuance. Acknowledgement of change in appearance but not seeing change as negative	Sexual or gender identity may influence treatment choice

*CSM* contralateral symmetrising mastectomy, *BM* bilateral mastectomy, *HCP* health care professional, *BC* breast cancer

#### Patient Motivations for Undergoing CSM

Two studies^31,32^ evaluated women’s motivation for undergoing CSM. Baker et al.^32^ found the two main motivations were to avoid a foreign body and to reduce the risk of complications (compared with reconstruction). Half of the patients in this study did not view their breasts as important for body image. Brown and McElroy^31^ identified motivations among sexual- and gender-minority (SGM) people, which included “political” motivation (to not hide the reality of breast cancer) and their lack of breasts aligning with sexual or gender identity.

#### Surgeons’ Attitudes Toward Surgery and the Theme of Flat Denial

Two studies^31,32^ identified a concept of flat denial, with some women experiencing biases of clinicians who did not support their decision to have bilateral mastectomy. Baker et al.^32^ found that 20.7% of women felt their surgeon did not support their decision to go flat. Some patients also noted assumptions based on gender (or sexual/gender identity), for example, the expectation to want reconstruction or presumed future regret caused by going flat.^31,32^

## Discussion

To our knowledge, this is the first systematic review to focus specifically on summarizing the available evidence regarding the clinical and patient-reported outcomes for women with unilateral breast cancer undergoing BM in the absence of a high genetic breast cancer risk who elect not to undergo breast reconstruction. The data consistently suggest that women who chose to undergo bilateral simple mastectomy are highly satisfied with their decision. Complication rates after bilateral surgery may be slightly higher than after unilateral mastectomy, but are significantly lower than after breast reconstruction, consistent with the findings of previous reviews.^34^

A further advantage of CM over reconstruction for symmetry is that women are less likely to require revision surgery over time, making CSM a much less costly option. The impact of BM without reconstruction on body image, sexuality, and quality of life, however, is less clear and requires further exploration.

This review highlighted issues associated with “flat denial” among clinicians and the resultant impact on patients’ experiences and outcomes. It is likely to be clinicians’ lack of appreciation for being flat as a valid option after mastectomy that underpins the current requirements that many centers have for patients to undergo psychological assessment if seeking CSM. Patients report feeling stigmatized by this request and may have to “battle” with clinicians to access the right surgery for them.^35,36^ Unsurprisingly, flat denial was shown have an adverse impact on patient satisfaction. Surgeons who do not accept “going flat” as a valid option may lack skills to create an aesthetic flat closure, or may intentionally leave excess tissue against the patient’s wishes in case they want reconstruction later.^33^

Rates of flat denial are likely be variable across breast units, but are reported to be lower among female surgeons and those with an exclusive breast practice.^32,33^ Both informed decision-making and good cosmesis improve satisfaction,^37^ but surveys show women are often not given the option to “go flat” and report non-aesthetic flat closures with extra skin, dog ears, and asymmetry after mastectomy.^35^

A further factor influencing women’s experiences is clinicians’ misunderstanding of their motivation for seeking CM, with surgeons often wrongly presuming that this procedure is sought for risk reduction. One study directly compared the views of health care professionals (HCPs) and women with breast cancer undergoing surgery and showed significant disparities in perceptions and views. Although almost all patients stated that their decision was motivated by a desire for an improved aesthetic outcome, more than half of HCPs perceived fear of recurrence as the main driver for surgery.^38^ This is consistent with the findings of our current review. Even clinicians who are supportive of women seeking CSM express concerns about offering the procedure, either due to concern over increased complications or colleagues’ attitudes towards CPM.^14^

Mislabeling CSM as “prophylactic” surgery causes further issues because “risk-reducing” procedures are not recommended for women at population breast cancer risk due to a lack of oncologic benefit.^39^ This adds further to clinicians’ reluctance to discuss this option with patients. Indeed, in one study, fewer than one in six (16%) UK HCPs reported always initiating discussions about CM. In this study, clinicians also reported wanting to learn more about how to discuss options with patients, with 80% stating that they would undergo formal teaching if this was offered.^14^ This demonstrates a gap in the collective knowledge of HCPs discussing appropriate surgical options with women and an opportunity to bridge it, overcoming a barrier to the equitable provision of CSM.

Inconsistent patient-reported outcomes after breast surgery have been noted in other reviews, with little evidence suggesting differences between procedure types.^37,40^ Many women feel comfortable without reconstruction and are distressed by the remaining breast and resultant asymmetry, which can interfere with physical activity.^37^ Women’s self-image may change so that living flat is more congruent^36^ and does not affect femininity.^41^ Furthermore, in research by Brown and McElroy,^31^ the impact of cancer itself and medical treatment was felt by some to have a greater impact than surgery on patient-reported outcomes. This highlights the importance of patient-centerd care and the need to provide a range of surgical options so women can make informed decisions.

Although this review summarized the available evidence, it had several limitations. First the included studies were heterogeneous and used a variety of outcome measures. This made it difficult to directly compare studies or pool data. It also was difficult to identify women undergoing CM specifically for symmetry because the intent of surgery was often not reported. Only two studies included qualitative data, with patients’ views reported verbatim. Many studies made vague comparisons across procedure groups, making it difficult to determine which subgroups were being compared. This reduced the amount of data available for the review.

Several studies also included women at high genetic risk within the bilateral mastectomy group. These women may have been elected to have surgery for different reasons, so their perceptions of outcome may not have been comparable.

The total number of women who had CSM overall was small, and the findings were often from subgroup analyses because women seeking CSM were not specifically recruited as a population of interest for these studies. Almost all the studies reported outcomes for non-ethnically diverse women from North America (14 studies were based in the United States and 1 in Canada), limiting generalizability. The results of the review should therefore be interpreted with caution, but summarizing the available data is a first important step to improving access to care and outcomes for women seeking CSM in the future.

This review demonstrated a lack of high-quality evidence to support the provision of CSM as an alternative to breast reconstruction and highlighted the need for more research. Factors influencing flat denial and the current inequity in access to care need further exploration, and qualitative interviews with patients and clinicians would be the first step to exploring and addressing these challenges.

Symmetry after breast cancer surgery is important to many women, and health care professionals should offer patients fully informed choice about all options available, including CSM. Equity in access to care is also vital, and national guidelines for offering CSM co-developed with all key stakeholders including patients, surgeons, psychologists, and specialist nurses in collaboration with the professional associations and charities may assist in standardizing care and improving access for women seeking this option. Studies specifically evaluating long-term patient-reported outcomes for women electing to be symmetrically flat also are vital in providing data to help women make fully informed decisions about their options and in supporting the ongoing provision of care.

## Conclusions

Contralateral mastectomy is a safer alternative to breast reconstruction for women seeking symmetry, and contrary to clinicians’ concerns, decisional regret for bilateral mastectomy is low. Education for surgeons is necessary to prevent flat denial and facilitate patient-centered care. Further research including the development of an evidence-based care pathway will be essential to ensure that all women have equitable access to the full range of surgical options for symmetry including CSM so they can move forward with their lives in the way that suits them best.
